# Establishment of a 96-well transwell system using primary human gut organoids to capture multiple quantitative pathway readouts

**DOI:** 10.1038/s41598-023-43656-z

**Published:** 2023-09-29

**Authors:** Charles W. Wright, Naomi Li, Lynsey Shaffer, Armetta Hill, Nicolas Boyer, Stephen E. Alves, Sriraman Venkataraman, Kaustav Biswas, Linda A. Lieberman, Sina Mohammadi

**Affiliations:** 1 Discovery Immunology, Merck & Co., Inc., Cambridge, MA 02141 USA; 2 Quantitative Biosciences, Merck & Co., Inc., Boston, MA 02115 USA; 3 Discovery Chemistry, Merck & Co., Inc., Boston, MA 02115 USA; 4 Present Address: Moderna, Inc., Cambridge, MA USA

**Keywords:** Cell biology, Developmental biology, Drug discovery, Immunology

## Abstract

Disruptions in the gut epithelial barrier can lead to the development of chronic indications such as inflammatory bowel disease (IBD). Historically, barrier function has been assessed in cancer cell lines, which do not contain all human intestinal cell types, leading to poor translatability. To bridge this gap, we adapted human primary gut organoids grown as monolayers to quantify transcription factor phosphorylation, gene expression, cytokine production, and barrier function. In this work we describe and characterize a novel 96-well human gut organoid-derived monolayer system that enables quantitative assessment of candidate therapeutics. Normal human intestine differentiation patterns and barrier function were characterized and confirmed to recapitulate key aspects of in vivo biology. Next, cellular response to TNF-α (a central driver of IBD) was determined using a diverse cadre of quantitative readouts. We showed that TNF-α pathway antagonists rescued damage caused by TNF-α in a dose-dependent manner, indicating that this system is suitable for quantitative assessment of barrier modulating factors. Taken together, we have established a robust primary cell-based 96-well system capable of interrogating questions around mucosal response. This system is well suited to provide pivotal functional data to support translational target and drug discovery efforts.

## Introduction

Inflammatory bowel disease (IBD) is a collection of disorders that present as inflammation of the intestinal mucosa^1,2^. A hallmark feature of IBD is the loss of epithelial barrier function^3–5^. The gut epithelium plays two roles in homeostasis: while the epithelium prevents the leakage of luminal content into serosal spaces, it also serves as a conduit, relaying signals to immune cells in the lamina propria^5^. In IBD the loss of barrier function leads to dysregulated exposure of immune cells to gut luminal content. Additionally, cytokines and chemokines produced by the epithelium (e.g. IL-8, MCPs, IL-1β) exacerbate mucosal inflammation. A key driver in IBD is TNF-α. This cytokine, produced by macrophages, dendritic cells, and T cells plays a pleiotropic role in disease. Importantly, TNF-α causes pronounced barrier dysfunction^6,7^. Because of the central role for TNF-α, TNF-α-neutralizing therapeutics have been used for decades to treat IBD. However, while initially anti-TNF-α therapeutics are effective, subsets of IBD patients stop responding to these therapies, highlighting the need to develop a better understanding of the role for TNF-α in IBD. The development of translatable in vitro systems that quantitatively capture multiple TNF-α signaling outputs would be pivotal in direct investigation of physiologically relevant biology and enable development of more effective therapeutics.

Current high-content gut barrier function models are based on human adenocarcinoma cell lines Caco-2 and HT-29^8–10^. A considerable deficit of these models is the lack of cell type complexity found in the human intestine. Four predominant cell types are found in the human intestine: absorptive enterocytes, secretory goblet, enteroendocrine, and Paneth cells^11^. These cell types carry out the functions of the intestine, thus are important constituents of in vitro barrier function cell-based assays. As cell line-based systems do not contain multiple cell types, gut organoid-based systems have been adapted to interrogate barrier function.

Gut organoids are complex cultures that contain most of the cell types found in the human intestine^12,13^. These cultures have been used to recapitulate important aspects of human disease *in vitro*^14–16^. An important challenge with organoid cultures is access to both apical and basolateral surfaces of the epithelium. The basolateral surface faces the culture media in organoids grown in 3D, thereby limiting access to the apical surface. Additionally, embedded growth in hydrogels (such as Matrigel), may limit access of treatment compounds or biologics to the basolateral surface. These challenges highlight the need for developing robust systems in which access to both apical and basolateral surface is not hindered by polarity or hydrogels. To this end, human gut organoid-derived cultures have been adapted to 2D monolayer cultures and used to model a range of biology from infectious diseases to barrier function defects^17–21^. While these systems add value for modeling human gut biology, implementation of multiple quantitative pathway readouts has not been demonstrated.

In this work, we describe the development and characterization of a robust 96-well human gut organoid-derived monolayer system. We show the implementation of multiple readouts in this system including quantitative measures of transcription factor phosphorylation, gene expression, cytokine production, and barrier function. We focus on TNF-α-mediated signaling events and show pathway modulation by quantifying the effects of TNF-α inhibitors using multiple assays. This miniaturized system enables testing of candidate therapeutics in a complex and physiologically relevant culture system that recapitulates key aspects of the human intestine. These include barrier function, polarization, and production of inflammatory cytokines.

## Results

### Establishing and characterizing organoid-derived monolayer cultures

**Figure 1:**
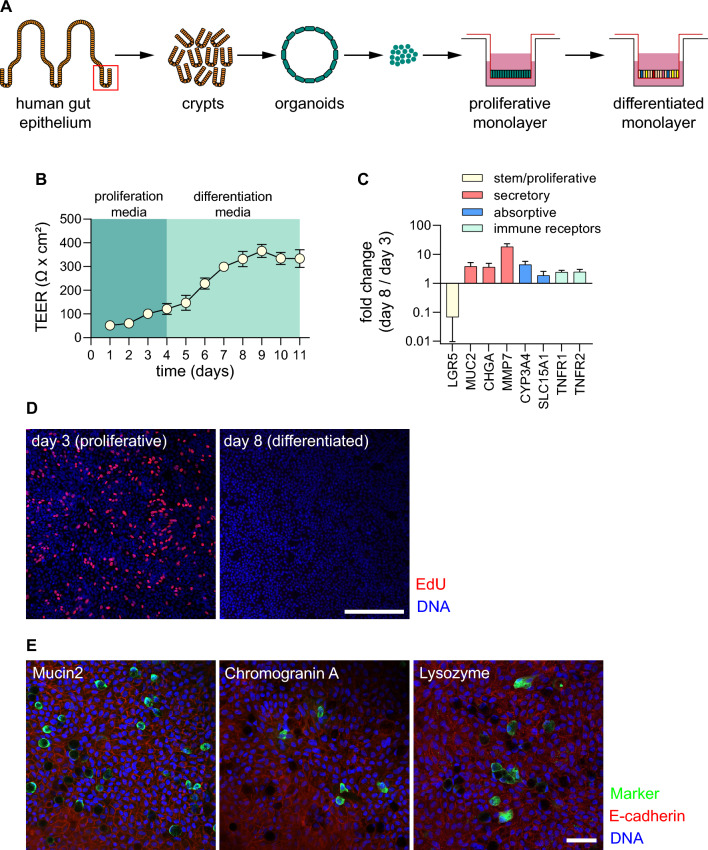
Establishment and characterization of organoid-derived monolayer cultures. (**A**) Schematic of 3D organoid generation and adaptation into 2D organoid-derived monolayers. Stem cell enriched, 3D organoids were generated from crypts isolated from healthy human intestinal epithelial tissue. These organoids were broken into single cells and seeded onto semi-permeable transwells to generate 2D cultures. (**B**) TEER measurements over a proliferation and differentiation time course. Cultures were grown in 96-well transwells in proliferation medium for 4 days before switching to differentiation medium for the following 7 days. Barrier function was measured in raw resistance values and normalized by surface area to obtain TEER values. Measurements were taken at approximately 24 h intervals after seeding for the duration of this culture. Each data point is an average of 8 wells. (**C**) Gene expression analysis for markers of stem/proliferative (yellow), secretory (red), and absorptive (blue) cells, as well as TNF receptors (green). Values are reported as fold change in differentiated cultures compared to proliferative cultures. Cultures were grown in 96-well transwells and each data point is an average of 8 wells. (**D**) Staining for S-phase cells using EdU assay for proliferative (day 3) and differentiated (day 8 cultures). Cultures were grown in 12-well transwells for imaging. Pseudocoloring is as follows: blue, DNA; red, proliferative/S-phase cells. Scale bar = 200 µm. (**E**) Differentiated organoid-derived monolayers were fixed and stained for makers of secretory cell types. Pseudocoloring is as follows: blue, DNA; green, lysozyme, mucin 2, or chromogranin A; red, E-cadherin. Scale bar = 50 µm.

Human adult stem cell (ASC) gut organoid cultures were established from the ileum and maintained as previously described^22,23^ (Fig. 1A). Organoid-derived monolayer cultures were established by adapting a previously published protocol^24^ to human ASC organoids. Notably, 96-well transwell cultures were used to facilitate implementation of automation, thereby increasing throughput, and enabling dose-curve experiments. Importantly, commercially available reagents and robots were used to facilitate ease of implementation in any lab setting. Briefly, 3D organoid cultures were dissociated to single cells and plated onto 96-well transwells (Fig. 1A). Cultures were allowed to differentiate by withdrawing Wnt pathway agonists from culture medium (see “Methods” for details). Monolayer cultures were subsequently characterized for barrier function and differentiation. Barrier function was quantified by measuring transepithelial electrical resistance (TEER), a non-invasive measure of barrier integrity that allowed for longitudinal measurements on the same transwells. This method showed that cultures typically reached a plateau of barrier tightness ~ 8 days after seeding (Fig. 1B). Importantly, high TEER readings were maintained, enabling multi-day experiments.

The differentiation status of cultures was determined through assessing presence of functional cell marker through gene expression analysis and immunofluorescence microscopy. Gene expression analysis in cells grown in 96-well transwells showed that LGR5, a marker of adult stem cells in the intestine was downregulated as cultures differentiated (Fig. 1C), suggesting that proliferative cells were transitioning to differentiated cells during the course of the experiment. Importantly, the expression of secretory and absorptive cell markers (secretory: MUC2, CHGA, MMP7; absorptive: CYP3A4, SLC15A1) were upregulated in differentiated cultures, indicating the presence of terminally-differentiated cells (Fig. 1C). Additionally, we showed that gene expression patterns were similar when comparing cells grown in 96-well and 24-well transwells (Fig. S1A). Concomitantly, differentiated cultures showed a marked reduction in S-phase cells (Fig. 1D), indicating the presence of terminally differentiated cells. Furthermore, staining for functional cell markers using immunofluorescence microscopy confirmed the presence of goblet cells, enteroendocrine cells, and Paneth cells (Fig. 1E). Taken together, these results indicate that monolayer cultures differentiate to form the expected intestinal cell types while maintaining barrier function.

Well-to-well variability in developmental gene expression was assessed next. Edge effects in miniaturized cell culture systems could skew observations. Developmental gene expression was assessed across a 96-well transwell plate: 16 wells were examined at a proliferative state (day 3 after seeding) and 80 wells were examined at a differentiated state (day 8 after seeding and day 4 after differentiation—see Methods section for details). While some variability in gene expression was observed across the plate, distinct patterns such as edge effects could not be identified (Fig. S1B). To mitigate potential noise in the 96-well transwell system additional replicates (wells) were included in downstream assays (Fig. S2).

### Developing a 96-well transwell TNF-α-based barrier damage system

**Figure 2:**
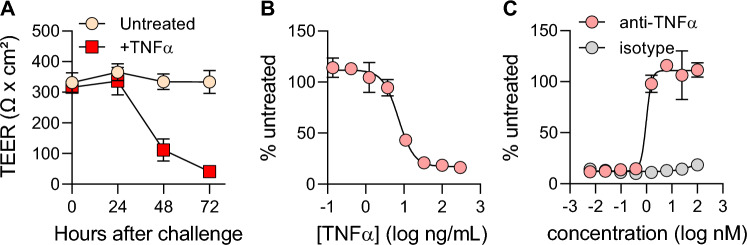
Developing a 96-well transwell TNF-α-based barrier damage system. (**A**) Barrier function was quantified in untreated monolayers (yellow) or monolayers challenged with 40 ng/mL TNF-α (red). At the time of treatment initiation, media on both apical and basolateral sides of the transwell was changed to media containing TNF-α. TEER measurements were taken immediately before challenge and then in 24-h intervals. Each data point represents the average of 8 cell culture replicates. (**B**) Barrier disruption following 72-h challenge with an 8-point dose curve with TNF-α. Values are reported as %untreated, which was obtained by averaging 5 cell culture replicates for each concentration of TNF-α and normalizing to the average TEER value of untreated controls (4 cell culture replicates). (**C**) Evaluation of anti-TNF-α antibody for the prevention of barrier damage. Monolayers were pre-treated for 1 h with 8-point dose curves of either anti-TNF-α neutralizing antibody or an isotype-matched control before challenge with 40 ng/mL TNF-α. TEER measurements from 5 wells per condition were taken at 72-h post-challenge and plotted in a similar manner to (**B**). All data in this figure were collected from cultures grown in 96-well transwells.

As TNF-α is a key driver of barrier dysfunction in IBD^6,7^, barrier damage by TNF was assessed using ASC organoid-derived monolayer cultures. Importantly, gene expression analysis showed that TNF receptors were expressed in differentiated 96-well transwell cultures (Fig. 1C). Quantification of barrier over time showed that the addition of TNF-α to culture media on both apical and basolateral surfaces resulted in a gradual reduction in TEER (Fig. 2A). At 24 h post TNF-α addition, little loss in TEER was observed. However, at 48- and 72-h post-treatment, a marked reduction in TEER was recorded. Next, the effect of a dose series of TNF-α upon barrier function (TEER) was quantified. This experiment generated a dose-dependent reduction in TEER, measuring a half maximal effective concentration (EC50) of 7 ng/mL (Fig. 2B). These results demonstrate that this ASC organoid-derived monolayer system is responsive to TNF-α and could be used to evaluate TNF-α pathway modulators.

A robust plate map was developed to incorporate both positive (with TNF-α added) and negative (no treatment) controls on each plate analyzed (Fig. S2A) in order to enable quality control calculations for each experiment. Analysis of a series of independent experiments showed a robust assay window, comparing no treatment and TNF-α treated wells (Fig. S2B and C). Effect size was quantified using a Z’ calculation. The average Z’ value was 0.65 (Fig. S2D), indicating the robustness of the assay in 96-well transwells.

The effect of TNF-α pathway modulators upon barrier function was quantified next. TNF-α-targeting biologics have revolutionized treatment for IBD over the last two decades^25,26^ so the effect of an anti-TNF-α monoclonal antibody was assessed. Addition an anti-TNF-α antibody to 96-well monolayer cultures treated with TNF-α efficiently rescued barrier damage in a dose-dependent manner (Fig. 2C). The EC50 for the anti-TNF-α antibody was measured to be 1 nM, indicating robust inhibition of TNF-α-mediated damage. Importantly, an isotype antibody used at the same concentrations failed to rescue barrier damage, indicating that this phenotype is specific to the anti-TNF-α antibody activity. These results indicate that our organoid-derived monolayer system could be deployed to quantitatively assess the effectiveness of TNF-α pathway modulators.

### Apical versus basolateral TNF-α signaling

**Figure 3:**
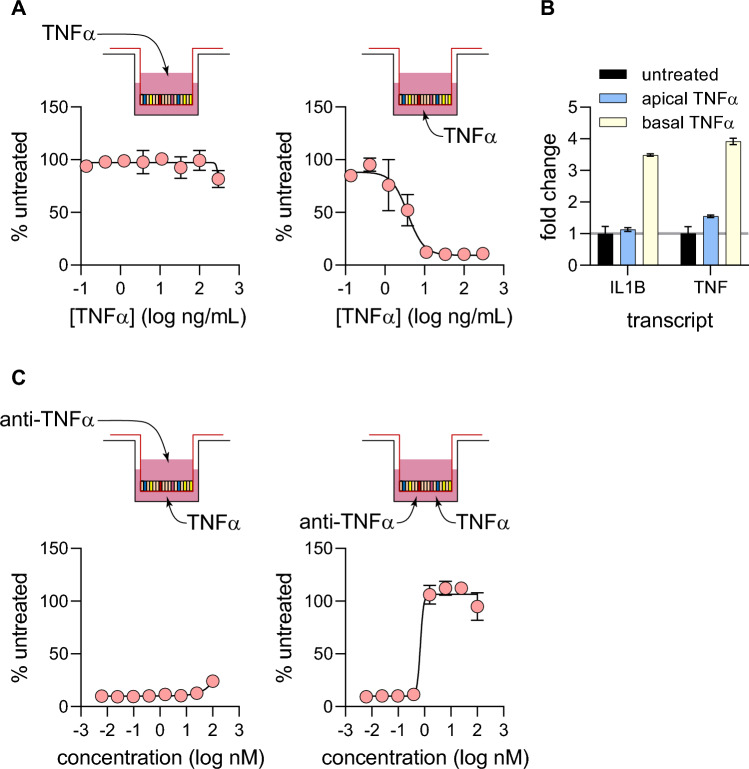
Apical vs basolateral TNF-α signaling. (**A**) Monolayers grown in 96-well transwells (5 wells per condition) were challenged with an 8-point dose curve of TNF-α on either the apical or basolateral sides of the monolayer. TEER measurements were taken at 72-h post-challenge and analyzed in a similar manner to Fig. 2B. (**B**) Quantifying differences in transcriptional response to 40 ng/mL TNF-α challenge on either side of monolayers. 6 h post-challenge, monolayers grown in 24-well transwells (3 wells per condition) were lysed and processed for qPCR. Gene expression for IL1B and TNF were quantified, and fold change values were calculated by normalizing to untreated controls. (**C**) Quantifying the potency of anti-TNF-α neutralizing antibodies by different routes of delivery. Monolayers grown in 96-well transwells (5 wells per condition) were pre-treated with anti-TNF-α antibody on either apical or basolateral sides before TNF-α challenge on the basolateral side. TEER measurement was taken 72 h post-challenge and EC50 values and curve fit was performed in the same manner as Fig. 2C.

Next, we investigated the polarity of TNF-α signaling. Our monolayer system allows for access to both apical and basolateral surfaces of the epithelium, thereby enabling us to parse differences in the location from which signaling is initiated. Monolayer cultures were challenged with TNF-α (as described in Fig. 2), however here TNF-α was added either to the apical or basal chamber of 96-well transwell cultures. Barrier integrity was then quantified using TEER. While apical challenge with TNF-α did very little damage to the barrier, basolateral challenge reduced TEER in a dose-dependent manner (Fig. 3A). The EC50 for basolateral TNF-α challenge was measured to be 4 ng/mL, a figure very similar to that observed with TNF-α challenge on both sides of the epithelium (7 ng/mL, Fig. 2B). This observation indicates that most of TNF-α signaling is initiated in the basolateral compartment. These results were supported by gene expression analysis. The expression of well characterized TNF-α-responsive genes IL1B and TNF were quantified using quantitative PCR, showing that gene expression was stimulated only when TNF-α was added in the basal chamber of transwells (Fig. 3B).

Next, we investigated the directionality of barrier function rescue by anti-TNF-α antibody. TNF-α was added to the basolateral chamber and anti-TNF-α antibody was added either in the apical or basolateral chamber of 96-well transwell cultures. Addition of anti-TNF-α antibody to the apical chamber did little to rescue the barrier from damage by TNF-α (Fig. 3C). Conversely, addition of anti-TNF-α to the basolateral chamber rescued barrier damage in a dose dependent manner (EC50 = 0.7 nM; Fig. 3C), similar to results with antibody added to both chambers (EC50 = 1 nM; Fig. 2C). This experiment showed that the vast majority of signal seems to have initiated from the basolateral compartment in this culture system.

### Quantifying receptor-proximal phosphorylation events in organoid-derived monolayer cultures

TNF-α-induced barrier damage is a distal consequence of proximal events such as receptor binding and changes in gene expression and cytokine/chemokine secretion patterns. Therefore, we sought to develop a clear understanding of early events that shape eventual disruption of barrier function in organoid-derived monolayers. As a receptor-proximal event, phosphorylation of p65/RelA component of NFκB was quantified.

**Figure 4:**
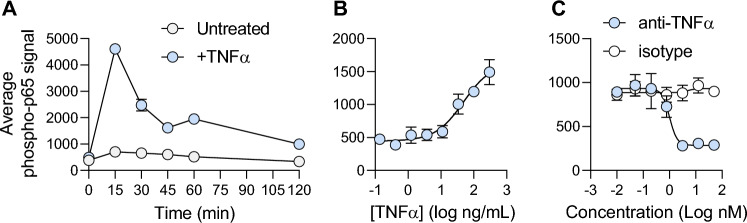
Quantifying p65/RelA phosphorylation in response to TNF-α stimulation of monolayer grown in 96-well transwells. (**A**) Organoid-derived monolayers grown in 96-well transwells (2 wells per condition) were left untreated or stimulated with 40 ng/mL TNF-α on both apical and basolateral surfaces. At indicated time points, cells were lysed and levels of phosphorylated p65 was quantified using an MSD assay. (**B**) A dose-series of TNF-α was added to both apical and basolateral surfaces of organoid-derived monolayers grown in 96-well transwells (4 wells per condition). At 15 min post stimulation cells were lysed and phospho-p65 was quantified. (**C**) Organoid-derived monolayers grown in 96-well transwells (3 wells per condition) were incubated with either an anti-TNF-α antibody or an isotype-matched control for 1 h. TNF-α (40 ng/mL) was added to all samples on both apical and basolateral surfaces and cells were harvested by lysis after 15 min of stimulation, followed by phospho-p65 quantification.

Upon receptor binding, TNF-α mediates NFκB pathway activation. A key molecular event in the activation of this pathway is the phosphorylation of p65 shortly after receptor binding. Phosphorylation is a labile event, so a time course experiment was designed to determine the peak phosphorylation for p65. TNF-α stimulation of 96-well transwell cultures led to a sharp increase in p65 phosphorylation, peaking at 15 min (Fig. 4A). After this peak, the signal slowly tapered off back to background levels by 120 min post-stimulation (Fig. 4A). Next, 96-well monolayers were stimulated with multiple TNF-α concentrations showing a dose-dependent increase in signal (EC50 = 48 ng/mL; Fig. 4B). Importantly, TNF-α-induced p65 phosphorylation could be inhibited by the addition of anti-TNF-α antibody (IC50 = 0.9 nM; Fig. 4C). These results indicate that in organoid-derived monolayers, receptor engagement by TNF-α leads to NFκB pathway activation through p65 phosphorylation.

To understand the extent by which organoid-derived monolayers can recapitulate inflammatory signaling, the same proximal target phosphorylation was performed in THP-1 cells, a known cellular model commonly used in IBD research due to their high reproducibility and ability to accurately simulate inflammation, notably for TNF-α signaling^27,28^. Consistently, we observed the strongest p65 phosphorylation at 15 min post TNF-α stimulation in THP-1 cells (Fig. S3A). Importantly, anti-TNF-α antibody decreased p65 phosphorylation in a dose-dependent manner, similarly to the organoid-derived monolayers (IC50 = 0.158 nM; Fig. S3B). Additionally, we confirmed the correlation of inhibiting p65 phosphorylation in organoid-derived monolayer cultures with MIP-1β inflammatory cytokine secretion, which is induced via TNFα-NF-κB signaling^29^, in the translational human whole blood assay (IC50 = 0.1514 nM, n = 11; Fig. S3C). Altogether, these data show robust transcription factor phosphorylation in response to TNF-α.

### Target gene expression in response to TNF-α stimulation

**Figure 5:**
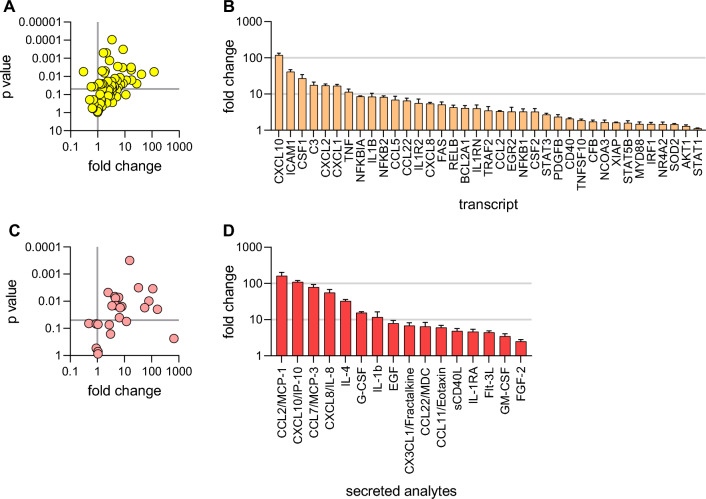
Target gene expression and cytokine production in response to TNF-α stimulation. (**A** and **B**) Assessing changes in NFκB-related gene expression following TNF-α stimulation. Following 6-h challenge with 40 ng/mL TNF-α on both apical and basolateral sides of the monolayer grown in 24-well transwells (3 wells per condition), a ratio of gene expression (by qPCR) compared to untreated controls was calculated for each marker in an 84-gene TaqMan array. Data were visualized as a volcano plot of p-value versus fold change (TNF-α-treated / untreated comparison) (**A**). Markers with a fold change above 1 and *p* < 0.05 were determined to be significantly upregulated and ranked by fold change (**B**). (**C** and **D**) Assessing changes in the cytokine secretion profile of organoid-derived monolayers following TNF-α challenge. Monolayers grown in 24-well transwells (3 wells per condition) were challenged with 40 ng/mL TNF-α on both apical and basolateral sides for 24 h before supernatant collection and Luminex analysis. Similar to gene expression analysis, results were visualized as a volcano plot of p-value vs fold change (**C**). Markers with a fold change above 1 and *p* < 0.05 were determined to be significantly upregulated and ranked by fold change (**D**).

The transcriptional response to TNF-α in organoid-derived monolayers was quantified next. Quantitative PCR was used to determine target gene expression profile. 2D organoid-derived 24-well monolayers were challenged with TNF-α for 6 h followed by RNA purification and quantitative PCR. Using a TaqMan array for the NFκB pathway, the expression of 84 NFκB related genes was quantified. Of the genes in this array, 68 were expressed in the monolayer system (Fig. 5A). Of these, 37 were upregulated significantly (*p* < 0.05) in the TNF-α-challenged cultures compared to untreated controls (Fig. 5B). Many TNF-α response and NFκB signaling pathway components were significantly upregulated, including proximal components of the TNF-α response (TRAF2, NFKBIA), transcription factors in the Rel/NFKB family (RelB, NFKB1, NFKB2), and death-inducing signaling complex (FAS). Additionally, expression of pro-inflammatory cytokines that directly affect the intestinal epithelium, TNF-α (TNF) and IL-1β (IL1B), was significantly upregulated. Furthermore, expression for many cytokines and chemokines associated with immune cell recruitment or differentiation were significantly upregulated, including IL-8 (CXCL8), IP-10 (CXCL10), CD54 (ICAM1), CSF1, C3, CXCL2, CXCL1, CCL5, MDC (CCL22), and MCP1 (CCL2) (Fig. 5B). These results indicate that TNF-α treatment led to robust transcriptional activation of NFκB signaling in organoid-derived monolayers.

### Cytokine production in response to TNF-α stimulation

An experiment was performed to analyze the cytokine secretion profile of organoid-derived monolayers in response to TNF-α. 24-well cultures were either untreated or challenged with TNF-α on the apical or basolateral side. Supernatants were collected from both apical and basolateral sides at 0, 3, 6, and 24 h after stimulation and analyzed using a 38-plex human cytokine/chemokine Luminex panel. Of the 38 analytes included, 28 were detected in monolayer cultures at 24 h (Fig. 5C). 16 cytokines or chemokines were significantly upregulated at 24 h in the supernatants that were collected in the basolateral side of the epithelium (Fig. 5D). The supernatants collected from the apical chamber were also evaluated but showed much lower or undetectable levels of almost all analytes. In agreement with the NFκB gene expression panel, many cytokines and chemokines associated with the recruitment and differentiation of immune cells were secreted in response to TNF-α, including CCL2/MCP-1, CXCL10/IP-10, CCL7/MCP-3, CXCL8/IL-8, IL-4, G-CSF, GM-CSF, CCL22/MDC, CX3CL1/fractalkine, and CCL11/eotaxin. These results showed that stimulation of organoid-derived monolayers with TNF-α produced a consistent secreted analyte signature. We next determined whether cytokine/chemokine secretion could be employed to evaluate TNF-α pathway modulators.

### Assessing TNF-α pathway modulators using IL-8 secretion

We showed that in response to TNF-α, organoid-derived monolayer cultures secrete a variety of cytokines and chemokines, modelling the inflammatory cascade that occurs in IBD. Notably, IL-8 was shown to be consistently secreted into the basolateral chamber in response to TNF-α stimulation (Fig. 5D). IL-8 is a key factor in amplifying the inflammatory response in IBD as it is a potent chemoattractant of neutrophils^30^. The observed production of IL-8 in response to TNF-α stimulation is expected and further validates the physiological relevance of this organoid-derived monolayer system. Next, we sought to further characterize the IL-8 response to TNF-α stimulation with the goal of quantifying the effects of TNF-α pathway modulators upon IL-8 production in 96-well transwell cultures.

**Figure 6:**
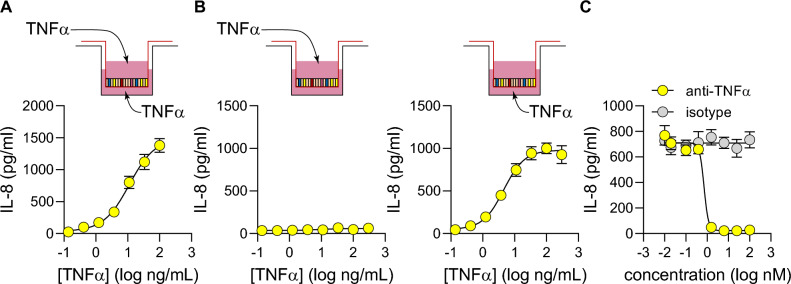
Assessing TNF-α pathway modulators by quantifying IL-8 secretion in monolayers grown in 96-well transwells. (**A**) Secretion of IL-8 into basolateral chamber was quantified after 24 h challenge with a dose range of TNF-α on both apical and basolateral surfaces of organoid-derived monolayers. (**B**) Secretion of IL-8 into basolateral chamber was quantified 24 h after stimulation on either apical or basolateral surface. (**C**) Organoid-derived monolayers were pre-treated for 1 h with either an anti-TNF-α antibody or isotype-matched control, then challenged with 40 ng/mL TNF-α on both apical and basolateral surfaces. IL-8 secretion was quantified in the basolateral chamber after 24 h. IL-8 amounts were quantified by ELISA, and each data point represents the average of 5 cell culture replicates. All curve fitting and potency values were generated using the same nonlinear regression model for the TEER measurements (Figs. 2 and 3). All cultures for this figure were grown in 96-well transwells and each data point represents the average of 4 wells.

First, organoid-derived monolayers were challenged with a dose curve of TNF-α and supernatants were collected from the basolateral chamber at 24 h and analyzed using an IL-8 ELISA. A dose-dependent increase in IL-8 secretion was observed (Fig. 6A), similar to the response observed in the TEER assay (Fig. 2B). EC50 values for the two experiments were similar (7 ng/mL for TEER and 11 ng/mL for IL-8 secretion) indicating concordance between assays.

A plate map was developed for IL-8 analysis, incorporating both positive (TNF-α-treated) and negative (no treatment) controls on each plate (Fig. S2E). Multiple independent 96-well transwell plates were analyzed, showing a consistent window (Fig. S2F and G) and an average Z’ value of 0.65 (Fig. S2H). These results indicate that IL-8 production in response to TNF-α is consistently robust in 96-well transwell cultures.

Next, the polarity of IL-8 production in response to TNF-α was determined. Monolayers were challenged with TNF-α from either the apical or basolateral side and IL-8 secretion quantified in the basolateral chamber. A dose-dependent IL-8 response was observed only upon TNF-α stimulation from the basolateral side of the epithelium (EC50 = 4.5 ng/mL; Fig. 6B). Once again, this readout recapitulates TEER results (Fig. 3A), as there was almost no signal observed with apical challenge with TNF-α. These results show that IL-8 secretion is a reliable indicator for TNF-α pathway activity, so the effect of pathway inhibitors was quantified next.

An anti-TNF-α antibody decreased IL-8 secretion levels in in TNF-α-stimulated monolayer cultures a dose-dependent manner (IC50 = 0.7 nM; Fig. 6C). This half maximal value was once again similar to results obtained with the TEER assay (EC50 = 1 nM; Fig. 2C). Taken together, these results indicate that IL-8 secretion is a dependable method to quantitatively assess the effect of TNF-α pathway modulators.

## Discussion

In this work we show the development of a 96-well transwell primary human culture system that recapitulates key features of intestinal inflammation. Efforts focused on incorporating multiple quantitative pathway readouts. We demonstrate that organoid-derived monolayer cultures are amenable to quantitative assessment through multiple readouts while retaining important physiologically-relevant features (e.g. cell composition, polarity). We first adapted organoids to a monolayer cultures system and demonstrated efficient differentiation and barrier function (Fig. 1). We showed that TNF-α reduced barrier function in a dose-dependent manner and that loss of barrier function could be rescued by the addition of TNF-α-pathway neutralizing antibodies (Fig. 2). As epithelial cells are polarized in the human intestine, unidirectionality of TNF-α signaling was investigated. TNF-α-mediated barrier damage could be initiated only from the basolateral compartment, demonstrating that epithelial cell polarization was preserved in our organoid-derived monolayers (Fig. 3). Additionally, we showed that key receptor-proximal signaling events (phosphorylation, target gene expression, cytokine/chemokine production) were intact and that these events could be used as quantitative readouts for receptor engagement (Figs. 4, 5, 6). Taken together, we have demonstrated that organoid-derived monolayer cultures are a tractable system to investigate gut inflammatory indications.

Importantly, we have established molecular and phenotypic readouts in a 96-well transwell format that span a large time scale after receptor engagement, from minutes (phosphorylation) to hours (cytokine/chemokine production) to days (barrier dysfunction). This range of quantitative readouts will enable future screening for candidate therapeutics that target the TNF-α pathway. This approach may be particularly pertinent to discovery of alternatives to anti-TNF-α therapies. A large fraction of IBD patients either do not respond to or lose responsiveness to anti-TNF-α therapies^31,32^. The system described in this work could be implemented to interrogate pathway components downstream of TNF-α as potential targets. Alternatively, the system could be used to screen for new targets important for the maintenance of barrier function with the eventual goal of supplementing or replacing anti-TNF-α therapies.

We have described a 96-well transwell system that is capable of quantifying the efficacy for anti-TNF-α therapeutics. Quantification of anti-TNF-α antibody was done in prophylactic mode: neutralizing antibody was added to cells first, then TNF-α was added. Implementing a system capable of evaluating therapies in a therapeutic mode (i.e. adding TNF-α first, followed by candidate therapeutics) would be highly impactful. To do this, a careful study must be undertaken to determine the exact timing for the addition of TNF-α. After a certain point in time, the barrier will be damaged beyond repair. Of note, we know that our cultures contain very few proliferative cells at the time of TNF-α challenge (Fig. 1D). This may be an issue if tissue repair is required after challenge. One could take advantage of the flexibility of the monolayer system described and adjust the differentiation schedule, allowing for the presence of a small number of proliferative/stem cells, which could repair a damaged epithelium after the removal of barrier disrupting agents. This would enable the implementation of a 96-well transwell system capable of quantitative evaluation in therapeutic mode.

Importantly, in addition to miniaturization and automated readouts, this platform reaches a mature (differentiated) state in one week. Most Caco-2 systems take considerably longer to mature after plating, and still do not recapitulate many key aspects of intestinal epithelial biology. Conversely, in addition to more rapid maturation, this organoid-derived platform can generate multiple functional cell types found in the intestinal epithelium, develop a physiological barrier function, and appropriately responds to inflammatory stimuli such as TNF-α.

To ensure robustness, positive and negative controls were included on each 96-well transwell analyzed (Fig. S2). Additionally, to address potential variability in readouts, multiple wells were included for each condition. Typically, 4 to 5 replicates were included in 96-well transwell assays, allowing for quantitative assessment of 16 treatment conditions per plate (in addition to controls). A few actions could be taken to reduce the number of replicates per condition, thereby analyzing more conditions per 96-well transwell. As we further develop this system, cell seeding and media changes could be automated to ensure more consistent handling of cultures. Furthermore, more rigorous environmental controls could be implemented. As an example, humidified culture plate lids could be used, similar to those used for higher cluster (e.g. 1536-well) plates.

A number of organoid-derived monolayer systems have been described recently^17–21,33–41^. These systems highlight the vast potential of primary epithelial cell systems to recapitulate various aspects of intestinal biology and disease. These range from investigating transporter function to pharmacokinetic studies, to co-culture of epithelial cells with pathogens and/or immune/stromal cells. While similar to the system described in our paper, previously published publications have not focused on teasing apart inflammatory signaling pathways using multiple quantitative readouts in a 96-well format. As of this writing, our work is the first demonstration of quantifying modulation of transcription factor phosphorylation, gene expression, cytokine secretion, and barrier function in response to a pro-inflammatory cytokine using a single unified culture system. The fact that these diverse cadre of readouts could be captured robustly using the same cells will reduce variability in outcomes, thereby enabling faster and more reproducible generation of results.

Previous publications point to potential future advancement opportunities for our monolayer system. Notably, our system is composed solely of epithelial cells. An important feature of intestinal inflammation is the cross-cell type communication, mediated by immune and stromal cells, which is an important feature of initiating and maintaining an inflamed state. Incorporation of immune and/or stromal cells into our system will steer the model even closer to recapitulating important aspects of human disease.

The 96-well monolayer system described in this work is physiologically-relevant, yet practical. All reagents, consumables, and equipment used are commercially available. The only specialized equipment used is a robot that measures TEER (REMS system produced by World Precision Instruments (WPI)). While this piece of equipment ensures consistent measurements across a 96-well transwell, TEER could be measured without automation. Hand-held electrodes compatible with 96-well transwell systems are produced (e.g. WPI EVOM system) that are much more cost effective than a stand-alone robot. The wide availability of consumables and equipment is a substantial advantage over some organ-on-chip platforms that may require specialized fabrication, complicated laboratory setups, and extensive expertise and effort to implement. This system was designed to recapitulate cell composition and key inflammatory responses of the human intestine in a format that is amenable to robust hypothesis generation and testing in a quantitative manner.

## Materials and methods

### ASC organoid culture and monolayer seeding

Adult stem cell (ASC) intestinal organoid cultures were established by isolating crypts from human ileum tissue as previously described^22–24^. Non-transplantable postmortem intestine tissue collected from anonymzed donors following local and federal regulations was used in this study. Tissues were collected from previously-confirmed organ donors according to United Network for Organ Sharing (UNOS) guidelines. All experiments described in this work were performed with one donor culture. These ASC organoids were maintained and expanded by passaging once per week in a proliferation medium (50% L-WRN conditioned media + Y27632 + SB431542^24^). Before seeding, transwells were coated with 0.35 mg/mL Matrigel (Corning 354234) diluted with ice cold DPBS (Gibco 14190144) for at least 1 h at 37°C. After coating time was complete, Matrigel solution was aspirated and transwells were washed once with room temperature DPBS before plating the cells. 2D organoid-derived monolayers were generated from these 3D cultures. On day 5 post-passaging, 3D organoids, grown in 15 uL Matrigel domes, were collected in serum-free washing media (Advanced DMEM/F12; Invitrogen 12634). Organoids were collected in a 15 mL tube (24 domes per tube) and triturated repeatedly to disrupt Matrigel, centrifuged at 900× *g* for 5 min, and media and most of pelleted Matrigel were aspirated. Single cell suspensions were generated by suspending organoid pellets in 1 mL TrypLE Express (Gibco 12605010) per 15 mL tube and incubated in a 37°C water bath for 15 min. 14 mL of proliferation media was added to each tube and cells were centrifuged at 500× *g* for 5 min. Cells were counted and suspended in proliferation media to 50,000 cells per well for 96-well transwells (MilliporeSigma PSHT004R5), 150,000 cells per well for 24-well transwells (Corning 3460), or 450,000 cells per for 12-well transwells (Corning 3470). Cells were added to the apical chamber of Matrigel-coated transwells in 100 uL, 200 uL, or 500 uL proliferation media for 96-, 24-, or 12-well transwells respectively. Cells were added to transwells using either multichannel pipets (96-well transwells) or hand-held electronic repeat pipets (12- and 24-well transwells). 300 uL, 600 uL, or 1.5 mL proliferation media was added to the basolateral chamber for 96-, 24-, or 12-well transwells respectively.

12-well transwells were used for all imaging experiments, 24-well transwells were used for initial gene expression and cytokine secretion analysis, and 96-well transwells were used for phospho-p65 quantification, gene expression, cytokine secretion, and all trans-epithelial electrical resistance (TEER) measurement analyses. In all formats, 0.4 um pore sizes were used, and the membrane material was either polyester (12- and 24-well) or polycarbonate (96-well). After seeding, organoid-derived monolayers were grown in proliferation media for 4 days before switching to differentiation media (5% L-WRN conditioned). Monolayers were then grown in differentiation media for 4 days before typically initiating experiments on day 8 after seeding. Media was changed every 1–2 days.

### TEER measurements

TEER measurements in 96-well transwells were performed using a commercially-available automated TEER measurement system (World Precision Instruments SYS-REMS) using an electrode set specially made for Millipore 96-well transwells (World Precision Instruments REMS-96). Raw resistance values generated by the system were corrected to TEER values by multiplying by transwell surface area (0.11 cm^2^). For conditions in which monolayers were challenged with TNF-α, TEER values were further normalized to untreated samples to generate “% untreated” values.

### TNF-α-mediated barrier damage assay

To assess TNF-α-mediated barrier damage, fully differentiated monolayers (day 8 post seeding, day 4 post differentiation) were challenged with a dose curve of TNF-α (R&D Systems 210-TA-100). Challenge with TNF-α was initiated by media change. Anti-TNF antibody (Invivogen htnfa-mab1) was evaluated by pre-treating for 1 h before TNF-α challenge. TEER measurements occurred immediately after challenge and in 24-h intervals thereafter, with the 72-h timepoint being the final data point.

### Transcriptional analysis

RNA was purified using either column-based methods (Qiagen RNeasy kit part no 74104) or automated bead-based methods using the KingFisher Flex system (Thermo 5400630) and the MagMAX™ mirVana™ Total RNA Isolation Kit (Thermo A27828). To quantify organoid-derived monolayer differentiation patterns, cultures were harvested for RNA purification and qPCR analysis at proliferative and fully differentiated timepoints (days 3 and 8 after plating, respectively). To evaluate gene expression changes in response to TNF-α, fully differentiated cultures were challenged for 6 h before harvesting for RNA purification and qPCR analysis. At each indicated time point culture media was removed from both apical and basal chambers of transwells and lysis buffer from either RNeasy or MagMAX kits was added directly to cells. Samples were typically frozen at − 80°C prior to RNA purification according to manufacturer directions. Purified RNA was reverse-transcribed into cDNA using SuperScript IV (Thermo 11756500) and the TaqMan™ Fast Advanced Master Mix (Thermo 4444557) was used to carry out qPCR. TaqMan assays used in this study are listed in supplemental Table 1. Ct values were normalized to endogenous controls using the comparative Ct method^42^ and presented as fold change of differentiated/challenged cultures over proliferative/untreated controls as described previously^22^. The “Human NFκB Signaling” TaqMan array (Invitrogen 4413255, design ID: RPGZE7K) was used to quantify expression of NFκB target genes.

### p65/RelA Phosphorylation assay

Cultures grown in 96-well transwells were challenged with TNF-α with or without pre-treatment of an anti-TNF-α antibody on both apical and basolateral sides. At indicated timepoints, cells were lysed by adding 10× lysis buffer directly to the apical chamber (lysis buffer: Cell Signaling Technology 9803) and frozen at -80°C. Quantification of NFκB p65/RelA phosphorylation was performed using a Phospho-p65 (Ser536) Whole Cell Lysate Kit (Meso Scale Diagnostics K151ECD) according to the manufacturer protocol.

### Immunofluorescence microscopy

For the detection of markers of functional cell types or junction proteins, fully differentiated organoid-derived monolayer cultures grown in 12-well transwells were fixed using 4% paraformaldehyde, permeabilized with 1% Triton X-100, and blocked with 5% BSA. Monolayers were then incubated overnight at 4°C with primary antibodies for markers of interest, followed by secondary antibody staining at room temperature for 2 h. A 5 mm biopsy punch (Integra 33–35) was used to remove a section of each membrane. Stained monolayers were mounted onto slides using mounting media (ProLong Gold, Invitrogen P36935) and microscopy was performed with a Zeiss LSM800 confocal microscope using either 20× (Fig. 1D) or 63× (Fig. 1E) lenses. To stain for proliferative cells, an EdU assay was performed according to the manufacturer’s protocol. All reagents are listed in supplemental Table 2.

### Cytokine secretion analysis

Supernatants were collected from the basolateral side of transwells in either 24-well or 96-well transwells (Figs. 5C, D and 6, respectively) at indicated timepoints after TNF-α challenge. Cytokine levels in supernatants were analyzed by 38 plex MILLIPLEX MAP Human Cytokine/Chemokine Magnetic Bead Panel (Millipore HCYTMAG-60K-PX38) adapted for use with 96-well DropArray plates and DropArray LT210 washing station (Curiox Biosystems). IL-8 secretion levels were quantified using an IL-8 ELISA kit (R&D Systems D8000C).

### Statistical analysis and cell culture replicates:

All error bars indicate standard deviation. Curve fitting and EC_50_ values were generated in GraphPad Prism using a 4-parameter nonlinear regression model. An unpaired t-test was used to calculate p values (Fig. 5). Culture format and replicate information is as follows: Fig. 1B: 96-well transwells, 8 wells per time point; Fig. 1C: 96-well transwells, 8 wells per condition. Figure 2: all 96-well transwells, 2A: 8 wells per condition, 2B: 5 wells per condition, and 2C: 5 wells per condition. Figure 3A: 96-well transwells, 5 wells per condition, Fig. 3B: 24-well transwells, 3 wells per condition, Fig. 3C: 96-well transwells, 5 wells per condition. Figure 4: all 96-well transwells, 4A: 2 wells per condition, 4B: 4 wells per condition, and 4C: 3 wells per condition. Figure 5: 24-well transwells, 3 wells per condition. Figure 6: 96-well transwells, 4 wells per condition.

### Supplementary Information

Supplementary Information.

## Data Availability

No ‘omics’ datasets were generated. The datasets generated in the current study are available from the corresponding author on reasonable request.
