# Supplementary material for: Establishment of a 96-well transwell system using primary human gut organoids to capture multiple quantitative pathway readouts
Source: Sci Rep. 2023 Sep 29;13:16357. doi: 10.1038/s41598-023-43656-z (PMC10541891; doi:10.1038/s41598-023-43656-z)
Supplement: Supplementary file 1 — Supplementary Information. [file 41598_2023_43656_MOESM1_ESM.pdf]

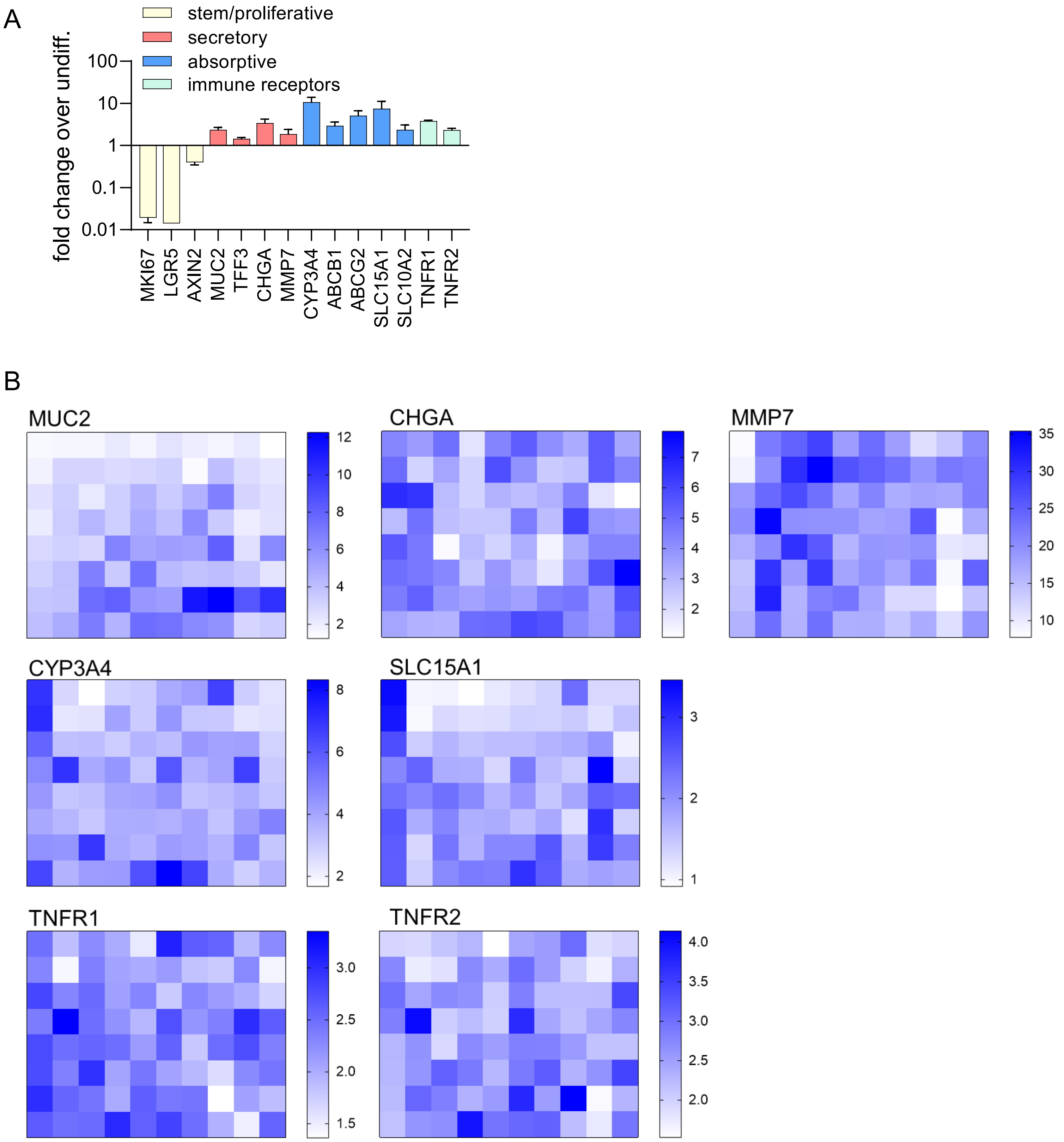
A
stem/proliferative
secretory
absorptive
immune receptors
fold change over undiff.
100
10
1
0.1
0.01
MKI67
LGR5
AXIN2
MUC2
TFF3
CHGA
MMP7
CYP3A4
ABCB1
ABCG2
SLC15A1
SLC10A2
TNFR1
TNFR2
B
MUC2
CHGA
MMP7
CYP3A4
SLC15A1
TNFR1
TNFR2

Figure S1

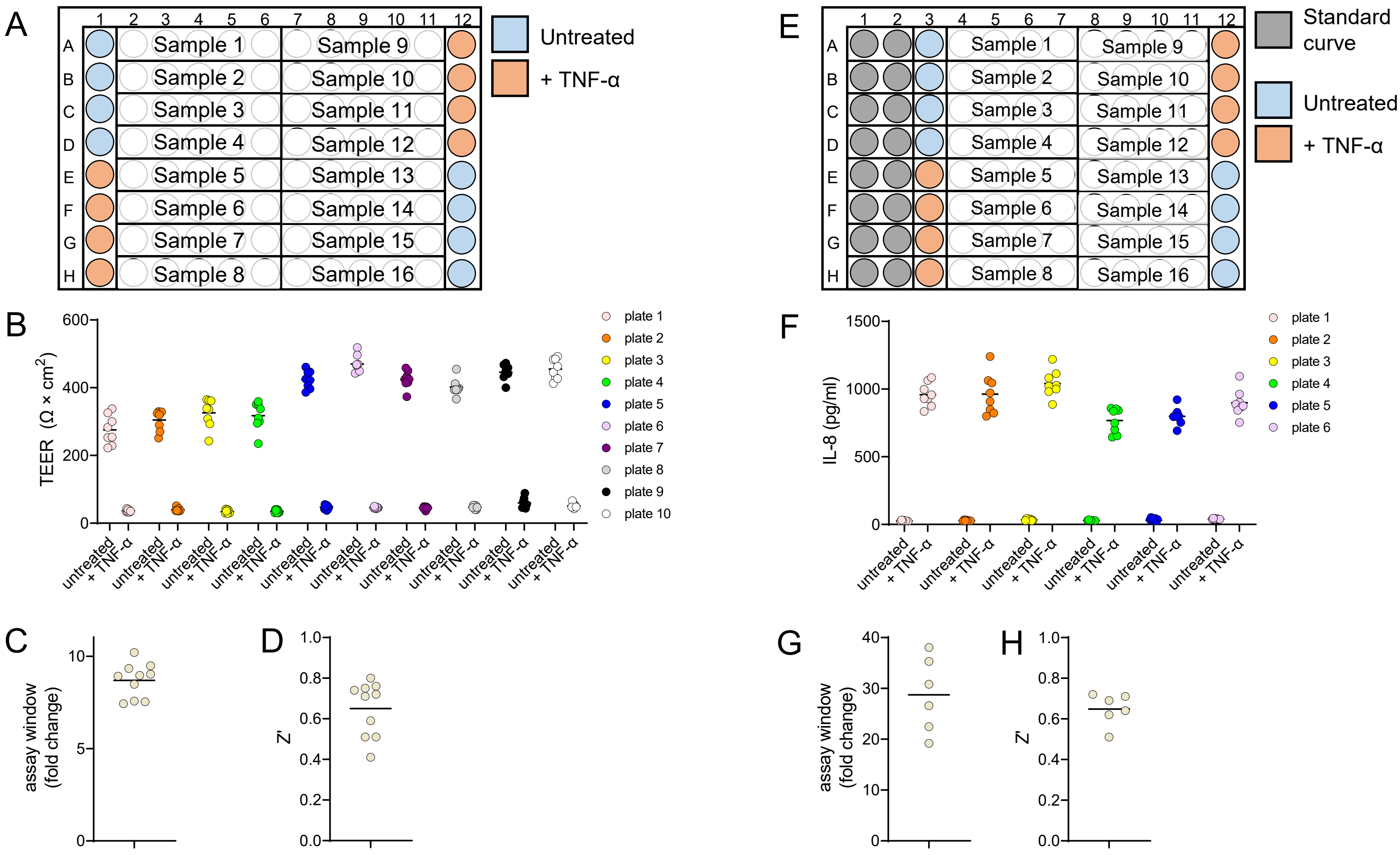
A
Sample 1
Sample 2
Sample 3
Sample 4
Sample 5
Sample 6
Sample 7
Sample 8
Sample 9
Sample 10
Sample 11
Sample 12
Sample 13
Sample 14
Sample 15
Sample 16
Untreated
+ TNF-α
B
TEER (Ω × cm²)
untreated
+ TNF-α
plate 1
plate 2
plate 3
plate 4
plate 5
plate 6
plate 7
plate 8
plate 9
plate 10
C
assay window (fold change)
D
Z'
E
Standard curve
Untreated
+ TNF-α
F
IL-8 (pg/ml)
G
assay window (fold change)
H
Z'

Figure S2

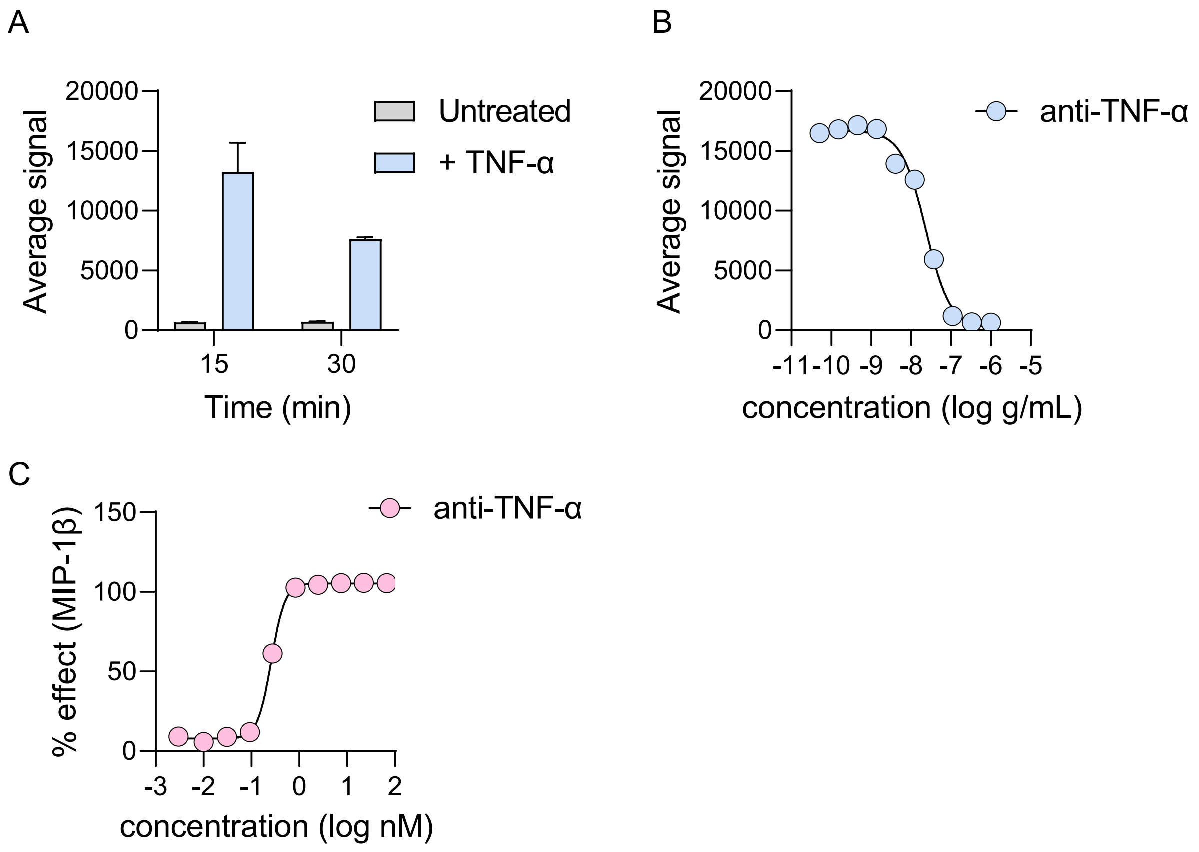
A
B
C
Average signal
20000
15000
10000
5000
0
15
30
Time (min)
Untreated
+ TNF-α
anti-TNF-α
-11
-10
-9
-8
-7
-6
-5
concentration (log g/mL)
% effect (MIP-1β)
150
100
50
-3
-2
-1
1
2
concentration (log nM)

Figure S3

Figure S1: Developmental gene expression analysis

(A) Gene expression analysis for cultures in 24-well transwells. Markers of stem/proliferative (yellow), secretory (red), and absorptive (blue) cells, as well as TNF receptors (green) were included. Values are reported as fold change in differentiated cultures (day 8) compared to proliferative cultures (day 3). Each data point is an average of 3 wells. (B) Well-to-well variability in developmental gene expression in 96-well transwells. Individual wells (80 wells) are represented using a heat map. Values are reported as fold change in differentiated cultures (day 8) compared to proliferative cultures (day 3).

Figure S2: Quantifying variability for TEER and cytokine secretion in 96-well transwells

(A) Plate format for TEER assays in 96-well transwells. 16 control wells were included on each plate analyzed: 8 wells with no treatment and 8 wells with 40 ng/mL TNF-α. Typically 5 replicates (wells) were included per test condition on each plate. (B) Plate-to-plate variability for TEER measurements. Data are presented for 10 different plates, analyzed over a period of months. Normalized TEER values are shown for each plate where each symbol represents a measurement from an individual well (8 no treatment wells and 8 TNF-α-treated wells). (C) The assay window for each plate was derived by calculating a ratio of TNF-α treated TEER values to no treatment TEER values. Average = 8.7 with range = 7.4 to 10.2. (D) Statistical effect size was determined by calculating a Z' value for each plate analyzed. Z' calculation is as follows:

$$Z' = 1 - \frac{3(\sigma_p + \sigma_n)}{|\mu_p - \mu_n|}$$

Where μ is the mean and σ is the standard deviation of both positive (p) and negative (n) controls. Average = 0.65 with range = 0.41 to 0.80. (E) Plate format for IL-8 analysis of 96-well transwell cultures. Both positive and negative controls were included on each plate. IL-8

quantification by ELISA required 2 columns of the analysis plates be dedicated to a standard curve, therefore only 4 replicates per condition were analyzed. (F) Plate-to-plate variability for IL-8 quantification. Data are presented from 6 independent plate, analyzed over a period of months. Absolute IL-8 levels (pg/mL) are shown for each plate where each symbol represents a measurement from an individual well (8 no treatment wells and 8 TNF-α-treated wells). (G) The assay window for each plate was derived by calculating a ratio of TNF-α treated IL-8 concentrations to no treatment IL-8 concentrations. Average = 28.7 with range = 19.2 to 38.1. (H) Statistical effect size was determined by calculating a Z' as described above. Average = 0.65 with range = 0.51 to 0.72.

Figure S3: Quantifying p65 phosphorylation and MIP-1β secretion in THP-1 and human whole blood

(A) THP-1 cells were untreated or treated with 10 ng/mL of TNF-α. Cells were lysed at the indicated timepoints, and phosphorylated p65 was quantified by MSD assay. (B) THP-1 cells were treated with a dose range of anti-TNF-α in the presence of 10 ng/mL of TNF-α. Phosphorylated p65 was quantified by MSD assay. (C) Human whole blood was treated with 10 ng/mL of TNF-α and dose-series of anti-TNF-α antibody. MIP-1β secretion was quantified by MSD assay.

Supplemental Table 1: TaqMan probes used in this study

transcript	TaqMan ID	marker type
PPIB	Hs00168719_m1	endogenous control
MKI67	Hs01032443_m1	proliferative/stem cells
LGR5	Hs00969422_m1	
AXIN2	Hs00610344_m1	
MUC2	Hs03005103_g1	secretory cells
TFF3	Hs00902278_m1	
CHGA	Hs00900370_m1	
MMP7	Hs01042796_m1	
P-gp/ABCB1/CD243	Hs00184500_m1	absorptive cells
BCRP/ABCG2	Hs01053790_m1	
SLC15A1	Hs00192639_m1	
SLC10A2/ASBT	Hs01001557_m1	
TNFR1/TNFRSF1A	Hs01042313_m1	immune receptors
TNFR2/TNFRSF1B	Hs00961750_m1	
IL1B	Hs01555410_m1	cytokines
TNF	Hs00174128_m1	

Supplemental Table 2: Fluorescence microscopy reagents

Reagent	Dilution	Source	Catalog #
Mouse anti-human Mucin 2 antibody	1:200	Dako	M731329-2
Rabbit anti-human Lysozyme antibody	1:500	Invitrogen	PA5-16668
Rabbit anti-human Chromogranin A antibody	1:100	Abcam	AB15160
Hoechst 33342	1:2000	Invitrogen	H3570
Goat anti-rabbit Alexa 488	1:250	Invitrogen	A-11034
Goat anti-mouse Alexa 594	1:250	Invitrogen	A-11032
Click-iT EdU Cell Proliferation Kit for Imaging, Alexa Fluor 594	N/A	Invitrogen	C10339
